# How does premenstrual syndrome affect occupational performance?

**DOI:** 10.1186/s12905-026-04286-5

**Published:** 2026-01-24

**Authors:** İbrahim Yavuz Tatlı, Gamze Kurt

**Affiliations:** 1 Department of Occupational Therapy, Faculty of Health Sciences, Kütahya Health Sciences University, Germiyan Campus, Kütahya, Türkiye; 2 Department of Occupational Therapy, Faculty of Health Sciences, Kütahya Health Sciences University, Kütahya, Türkiye

**Keywords:** Activities of daily living, Occupation, Premenstrual syndrome, Quality of life, Women

## Abstract

**Introduction:**

Premenstrual syndrome (PMS) is a condition that affects the quality of life and occupational participation of reproductive age women. The aim of this study was to investigate the relationship between PMS severity and occupational performance, meaningful time use, and quality of life and to compare these parameters between women with and without PMS.

**Methods:**

Fifty-three women with and without PMS were included in this cross-sectional research. Presence and severity of PMS, occupational problems, daily activity preferences, and quality of life were assessed by the Premenstrual Syndrome Scale (PMSS), Canadian Occupational Performance Measure (COPM), Modified Occupational Questionnaire (MOQ), and 12-Item Short-Form Health Survey (SF-12), respectively. The Spearman's rank correlation test was used for the relationship, and the Mann–Whitney U test and chi-square test were used for the differences between groups. A significance level of *p* < 0.05 was considered statistically significant.

**Results:**

Of the total 53 women involved in this study, 71.7% experienced PMS. For the women with PMS, there was a statistically significant positive correlation between the total duration of MOQ-obligatory activities and the MOQ-value (rho = 0.430, *p* = 0.007) and the COPM performance score (rho 0.330, *p* = 0.046). The results indicated that there was a significant negative correlation between the SF-12 physical and PMSS score (rho = -0.322, *p* = 0.048). A statistically significant difference was observed in PMSS (*p* < 0.001) and MOQ value (*p* = 0.019) scores, with women with PMS scoring higher than those without.

**Conclusion:**

Although no direct differences in occupational performance were observed between women with and without PMS, the time-use preferences and value perceptions of women experiencing PMS may influence occupational performance in distinct ways. Our findings suggest that maintaining a daily routine has a positive impact on performance among women with PMS, whereas increasing symptom severity is associated with a decline in quality of life.

## Introduction

Premenstrual syndrome (PMS), estimated to have a worldwide prevalence of 47.8%, is a condition that affects the quality of life of women of reproductive age. PMS emerges during the luteal phase of the menstrual cycle between days 14 and 28 post-ovulation and resolves within a few days of menstruation's beginning. The primary psychological and physical indications of PMS consist of irritability, depression, tearfulness, anxiety, difficulty concentrating, fatigue, abdominal bloating, breast tenderness, and headaches [6, 22].

Occupations are everyday activities that individuals, families, and communities engage in to occupy time and bring meaning and purpose to life. They include things people need to do, want to do, and are expected to do [32]. Obligatory occupations typically form part of the daily routine, such as activities of daily living, instrumental activities of daily living, and health management. In contrast, volitional occupations add value to time and encompass play, leisure, and social engagement [5].

PMS has a structure that affects occupational participation and disrupts routine and balance [25]. Previous research [7, 24] reported that premenstrual symptoms impact the daily life activities of women with PMS. Evidence from these studies indicates that factors including pain, fatigue, and mood alterations adversely influence occupational performance, social participation, and daily living among individuals experiencing PMS. Conducting an in-depth analysis of occupational differences between those with and without PMS could provide important insights for the development of novel therapeutic approaches. Sut and Mestogullari [29] stated that women with PMS had difficulty in performing daily work, family relations, social participation, and home-related work.

Analyzing the impact of symptoms on individuals' occupational preferences [9] and identifying the occupations that individuals consider important may be crucial for planning prevention and rehabilitation programs. Detailed activity analyses during the luteal phase of women of reproductive age will shed light on the impact of PMS on activity preferences and occupational performance. Furthermore, comparing the performance and preferences of women with and without PMS during this period will help to understand the differences. Research examining the effect of PMS on occupational performance is limited. Considering this gap in the literature, this study was planned to investigate the relationship between PMS severity and occupational performance, meaningful time use, and quality of life and to compare these parameters between women with and without PMS.

## Methods

### Study design and setting

This research was conducted from November 2023 to May 2024 as cross-sectional research. The study was approved by the Ethics Committee of Kütahya Health Sciences University (No: 2023/11–04) and all participants provided written informed consent. The research was carried out according to the principles of the Declaration of Helsinki.

### Participants

The inclusion criteria for this study in the study were being a woman between the ages of 18 and 35 and agreeing to participate voluntarily. Women who are pregnant or postmenopausal were excluded from the study. Announcements were made via social media, email and messaging groups for the research. Participants were recruited from among women who volunteered to take part in the study.

### Procedure

The required time intervals for administering the assessment scales were observed. Evaluations were performed face-to-face by the researchers within 5 days before menstruation. In these interviews, the COPM and MOQ were introduced to the participants with guidance provided according to the manual. The concepts of occupations, value, performance, and satisfaction included in the scale were explained to the participants during the interview. Activities in which participants were considered to experience performance and/or satisfaction problems, according to the scale, were recorded using the COPM. Participants completed the MOQ by self-reporting their activities over a 24-h period, based on recalling the previous day as a typical day. This procedure was conducted by the researcher İYT. Following this stage, responsibility for the subsequent assessments was assumed by another researcher.

In the subsequent phase of the interview, one of the researchers (GK) assumed responsibility for administering the PMSS and the Short Form-12 Health Survey SF-12 to the participants. These instruments, applied through a self-report method, were distributed to the participants for completion. During administration, the researcher provided guidance to participants who had questions regarding the forms.

The timing of menstruation was determined by examining participants' menstrual calendars and menstrual cycles (duration of bleeding and length of menstrual cycle). Participants whose menstruation occurred more than two days earlier or later than the expected date were excluded from the study in order to ensure accurate identification of the luteal phase [14].

### Data measurements

#### Premenstrual syndrome scale

The presence and severity of premenstrual syndrome in the participants were evaluated using the Premenstrual Syndrome Scale (PMSS) developed by Gençdoğan. PMSS requests participants to retrospectively evaluate their experiences, focusing on a period "during a week preceding menstruation". This 44-item scale has a five-point Likert rating (1: Never, 2: Very rarely, 3: Sometimes, 4: Often, 5: Always). The scale consists of 9 subscales including depressive affect, anxiety, fatigue, irritability, depressive thoughts, pain, appetite changes, sleep changes, and bloating. The lowest score that could be obtained from the whole scale is 44, and the highest score is 220. Participants with a total PMSS score of more than 50% are categorized as having PMS. Higher PMSS scores indicate more severe premenstrual symptoms [16].

#### Modified occupational questionnaire

The Modified Occupational Questionnaire (MOQ) was one of the assessment tools used in this study [27]. It is a measure of meaningful time use. Basically, the MOQ focuses on two important objectives: what the person was doing or how he/she used their time, and how the person felt about it. The MOQ is a time diary that asks respondents to report on what they do ‘‘on a typical day’’ in hour blocks from 05.00 to 05.00 the following day. The meaningfulness of each activity was investigated according to the following criteria: (a) category of activity, (b) reason why the person engaged in the activity, (c) value of the activity to the person and (d) perceived value of the activity to society.

There were three options to answer as to why the activity was performed: ‘‘I had to do it (obligatory),’’ ‘‘I wanted to do it (volitional),’’ or ‘‘I had nothing else to do (leisure).’’ Participants rate each activity’s meaningfulness on a 5-point scale (1 = not at all valuable, 5 = very valuable), taking into account both personal relevance and perceived societal importance [27]. This questionnaire aims to capture information on individuals’ daily routines by taking into account the temporal dimension and the degree to which activities are considered valuable and personally relevant. In the present study, categories b and c were employed to explore the reasons underlying participation in occupations and to evaluate the subjective value attributed to these occupations.

#### Canadian occupational performance measure

The Canadian Occupational Performance Measure (COPM) was one of the assessment tools used in this study [21]. It is an individualistic outcome measure implemented via a semi-structured interview. During the interview clients are asked by their occupational therapist to identify occupational performance problems within areas of their self-care, productivity and leisure. Using 10-point Likert scale clients rate each problem based on level of ‘importance’. The five most important problems chosen are rated, again based on self-perceived ‘performance’ and ‘satisfaction with performance’. Next, the occupational therapist then translates each client’s problem into a goal and plans interventions accordingly. During or at the end of therapy, the client’s perceived performance and satisfaction were re-evaluated. A change score can then be calculated by subtracting the post-treatment score from the initial score to determine of clinically significant gains or changes have been made [21]. The Turkish version of the Canadian Occupational Performance Measure (COPM-TR) has been validated and found to be reliable across different populations, demonstrating its suitability for both clinical practice and research purposes [31].

In this study, data from the COPM have been analyzed by calculating mean performance and satisfaction scores for the identified activities. Changes in these scores have been compared between participants with and without PMS to evaluate its impact on occupational performance. Statistical tests have been applied to determine whether differences in performance and satisfaction were significant.

#### 12-item short-form health survey

The Turkish version of the 12-Item Short-Form Health Survey (SF-12) was one of the assessment tools used in this study [28]. It was created by shortening the SF-36. It consists of two components named as Physical and Mental and scores for each component are calculated separately. The SF-12 Physical score is obtained from general health, physical functioning, physical role, and body pain sub-dimensions, while the SF-12 Mental score is obtained from social functioning, emotional role, mental health, and energy sub-dimensions. Both SF-12 Physical and SF-12 Mental scores range from 0 to 100, with higher scores representing better health. The X reliability and validity study of the scale was performed by [28].

### Statistical analysis

The data of this study was analyzed using the Jamovi (version 2.4.14) statistical analysis program. Continuous variables were characterized as means and standard deviations (mean ± SD) and categorical variables were presented as frequencies/percentages (n/%). The Shapiro–Wilk test was used to assess the normality distribution of the data. Due to the data not following a normal distribution, the strength and direction of the relationship between non-parametric variables were analyzed using Spearman’s rank correlation. This correlation coefficient ranges between −1 and + 1. Accordingly, a value below 0.19 indicates a very weak association, a value between 0.20 and 0.39 indicates a weak association, a value between 0.40 and 0.69 indicates a moderate association, a value between 0.70 and 0.89 indicates a strong association, and a value above 0.90 indicates a very strong association. Effect sizes were expressed as Spearman’s correlation coefficients, and 95% confidence intervals were calculated using Fisher’s r-to-z transformation [1]. The Mann–Whitney U test and chi-square test were utilized to compare women with and without PMS. A significance level of *p* < 0.05 was considered statistically significant. A post hoc power analysis was performed using G-Power (version 3.1.9.3) to evaluate the sufficiency of the sample size for the comparing SF-12 mental scores between women with and without PMS. Based on the observed effect size and group sample sizes, the achieved statistical power (two-tailed, α = 0.05) was calculated as 0.88, indicating that the study had sufficient power to detect the observed between-group difference.

### Positionality statement

#### Researcher İYT

With a background in occupational therapy and a personal interest in occupational participation and daily time use, my perspective is shaped by both academic training and lived experiences in occupation-centered clinical settings. My understanding of PMS reflects not only empirical literature but also direct observations of how cyclical health conditions affect daily functioning and occupational engagement. I was responsible for explaining and administering the COPM and MOQ in face-to-face interviews. My eight years of clinical practice and interest in activity patterns supported participants’ comprehension of these tools. By following standardized protocols, I aimed to reduce bias linked to my professional background and ensure valid interpretation.

#### Researcher GK

I identify as a cisgender woman and recognize that this positionality may influence the way I interpret participants’ narratives and prioritize certain aspects of occupational performance. While I do not experience severe PMS symptoms myself, I am aware of the diverse ways in which this condition manifests and the social stigmas that often accompany it. This awareness guided my commitment to creating a research environment that was empathetic, inclusive, and respectful of participants’ experiences.

Throughout the study, we remained reflexive about my assumptions and actively sought to minimize bias by incorporating participant feedback and consulting with peers from varied backgrounds. we view this research not only as an academic inquiry but also as a contribution to broader conversations about gendered health experiences and their implications for occupational science.

## Results

A total of 53 women participated in the study, of whom 71.7% (*n* = 38) experienced PMS, and the remaining 28.3% (*n* = 15) did not. The participants' age range in the study was 19 to 27, with a mean age of 21.9 ± 2.19 years. The demographic characteristics of the women are described in Table 1.

**Table 1:** Sociodemographic and clinical characteristics of the women

	Women with PMS (*n* = 38) (Mean ± SD)	Women without PMS (*n* = 15) (Mean ± SD)	*p*
Age *(years)*	21.8 ± 2.21	22.2 ± 2.18	0.512
BMI *(kg/m*^*2*^*)*	21.9 ± 3.62	21.4 ± 3.42	0.953
Age of menarche *(years)*	13.1 ± 1.20	12.9 ± 0.743	0.417
Menstrual cycle length *(days)*	28.5 ± 4.28	27.9 ± 2.03	0.473
Menstrual bleeding *(days)*	5.89 ± 1.18	5.60 ± 1.12	0.412
	n (%)	n (%)	p
Education level			0.713
*Bachelor’s degree*	32 (%60.4)	12 (%22.6)	
*Graduate degree*	6 (%11.3)	3 (%5.7)	
Income level			
*Income less than expense*	4 (% 7.5)	0 (% 0.0)	0.226
*Income equals expenses*	3 (% 5.7)	3 (% 5.7)	
*Income more than expense*	31 (% 58.5)	12 (% 22.6)	
Regular menstrual cycle			0.630
*Yes*	28 (%73.68)	12 (%22.6)	
*No*	10 (%26.31)	3 (%5.7)	
Gynecological disease			0.879
*Yes (PCOS)*	3 (%5.7)	1 (%1.9)	
*No*	35 (%66)	14 (%26.4)	
Chronic disease			0.263
*Yes (asthma, graves' disease, mediterranean anemia)*	3 (%5.7)	0 (% 0.0)	
*No*	35 (% 66.0)	15 (% 28.3)	
Smoking status			0.879
*Yes*	3 (%5.7)	1 (%1.9)	
*No*	35 (%66)	14 (%26.4)	
Alcohol using status			0.526
*Yes*	1 (%1.9)	0	
*No*	37 (%69.8)	15 (%28.3)	

*BMI* Body mass index, *PMS* Premenstrual syndrome, *n* Number of participants, *SD* Standard deviation

A statistically significant difference was found in PMSS (*p* < 0.001) and MOQ value (*p* = 0.019) scores, with women with PMS scoring higher than those without. However, women without PMS have statistically significantly higher SF-12 mental scores than women with PMS (*p* = 0.008). No significant differences were found in COPM performance (6.07 vs. 6.07; *p* = 0.872) or satisfaction (5.28 vs. 5.17; *p* = 0.785) between the PMS and non-PMS groups (Table 2).

- Spearman’s rank correlation analysis was conducted to examine associations among variables related to women with PMS. The analysis revealed a moderate positive correlation between the total duration of MOQ-obligatory activities and the MOQ-value (rho = 0.430, 95% CI [0.12, 0.65], *p* = 0.007). A weak positive correlation was also observed between the total duration of these obligatory activities and the COPM performance score (rho = 0.330, 95% CI [0.01, 0.59], *p* = 0.046).
- In contrast, the total duration of MOQ-voluntary activities showed weak negative correlations with both the MOQ-value (rho = − 0.372, 95% CI [− 0.62, − 0.06], *p* = 0.022) and the COPM performance score (rho = − 0.353, 95% CI [− 0.60, − 0.04], *p* = 0.032).
- Finally, significant weak correlations were found between the SF-12 physical score and the PMSS score (negative: rho = − 0.322, 95% CI [− 0.58, − 0.00], *p* = 0.048), as well as between the SF-12 physical score and the MOQ-value (positive: rho = 0.342, 95% CI [0.03, 0.60], *p* = 0.036) (Table 3).

**Table 2:** Comparison between women with and without PMS

	Women with PMS (*n* = 38) (Mean ± SD)	Women without PMS (*n* = 15) (Mean ± SD)	*p*
PMSS	145 ± 20.7	96.3 ± 13.1	< 0.001*
SF-12			
*Physical*	51.2 ± 7.51	53.8 ± 5.21	0.300
*Mental*	34.4 ± 7.92	41.6 ± 8.52	0.008*
COPM			
*Performance*	6.07 ± 1.73	6.07 ± 1.87	0.872
*Satisfaction*	5.28 ± 2.34	5.17 ± 2.06	0.785
MOQ			
*Value*	4.43 ± 0.378	4.19 ± 0.368	0.019*
*Obligatory Occupations (time)*	14.6 ± 5.82	12.3 ± 6.10	0.169
*Volitional Occupations (time)*	8.24 ± 5.15	10.7 ± 5.75	0.148
*Leisure time Occupations (time)*	0.853 ± 1.58	1.07 ± 2.12	0.852

*PMSS* Premenstrual Syndrome Scale, *COPM* Canadian Occupational Performance Measure, *SF-12* Short Form Health Survey, *n* Number of participants, *SD* Standard deviation

^*^p < 0.05

**Table 3:** Relationships between PMSS, COPM, SF-12 and MOQ scores of women with PMS

		PMSS	COPM-Performance	COPM-Satisfaction	SF-12 Physical	SF-12 Mental	MOQ-Value	MOQ-Obligatory	MOQ- Volitional
COPM-Performance	rho	−0.142							
	p	0.401							
COPM-Satisfaction	rho	−0.099	0.813						
	p	0.561	< 0.01*						
SF-12 Physical	rho	−0.322	−0.061	−0.206					
	p	0.048*	0.721	0.222					
SF-12 Mental	rho	−0.263	0.213	0.330	−0.203				
	p	0.111	0.205	0.046	0.221				
	p	0.880	0.949	0.519	0.757	0.308			
MOQ-Value	rho	−0.069	−0.043	−0.004	0.342	−0.008			
	p	0.682	0.940	0.981	0.036*	0.963			
MOQ-Obligatory	rho	−0.075	0.330	0.214	0.0150	−0.040	0.430		
	p	0.654	0.046*	0.204	0.368	0.814	0.007*		
MOQ- Volitional	rho	0.105	−0.353	−0.212	−0.136	0.014	−0.372	−0.946	
	p	0.530	0.032*	0.207	0.416	0.933	0.022*	< 0.01*	
MOQ-Leisure	rho	−0.050	0.114	0.256	−0.104	0.135	−0.334	−0.232	0.001
	p	0.780	0.521	0.145	0.558	0.448	0.053	0.186	0.993

*PMSS* Premenstrual Syndrome Scale, *COPM* Canadian Occupational Performance Measure, *MOQ* Modified Occupational Questionnaire, *SF-12* Short Form Health Survey

^*^p < 0.05

According to Spearman's analysis, it was found that significant negative moderate relationship between the MOQ value and the total duration of MOQ-leisure activities in women without PMS (rho = −0.581, 95% CI [− 0.85, − 0.07], *p* = 0.023). No statistically significant correlation was observed between the other parameters (Table 4).

**Table 4:** Relationships between PMSS, COPM, SF-12 and MOQ scores and subscale scores of women without PMS

		PMSS	COPM-Performance	COPM-Satisfaction	SF-12 Physical	SF-12 Mental	MOQ-Value	MOQ-Obligatory	MOQ- Volitional
COPM- Performance	rho	0.034							
	p	0.904							
COPM- Satisfaction	rho	−0.014	0.983						
	p	0.959	< 0.01*						
SF-12 Physical	rho	−0.264	−0.191	−0.170					
	p	0.341	0.496	0.544					
SF-12 Mental	rho	0.241	−0.107	−0.041	0.292				
	p	0.387	0.705	0.884	0.292				
MOQ- Value	rho	−0.316	0.212	0.263	−0.011	0.441			
	p	0.251	0.448	0.344	0.970	0.100			
MOQ- Obligatory	rho	0.119	0.475	0.469	0.017	0.262	0.415		
	p	0.673	0.074	0.078	0.952	0.346	0.124		
MOQ- Volitional	rho	−0.040	−0.338	−0.292	−0.277	−0.079	−0.090	−0.866	
	p	0.889	0.217	0.291	0.318	0.779	0.750	< 0.01*	
MOQ-Leisure	rho	−0.176	−0.481	−0.480	0.551	−0.050	−0.581	−0.492	0.082
	p	0.530	0.070	0.070	0.033	0.945	0.023*	0.063	0.771

*PMSS* Premenstrual Syndrome Scale, *COPM* Canadian Occupational Performance Measure, *MOQ* Modified Occupational Questionnaire, *MOQ-V* Value subscale, *SF-12* Short Form Health Survey

^*^p < 0.05

According to the COPM, the activities ranked first in terms of importance by the participants were recorded. The selection of these activities, identified as the most important within each group, was compared using the Chi-square analysis method. As result, there was no significant difference observed between the two groups in terms of choosing occupation (*p* = 0.512). Both groups identified a total of six different occupational categories while determining their most important occupations. The majority of women with PMS have chosen "activities of daily life" as their most important occupation, whereas women without PMS prioritize "education" activities (Table 5).

**Table 5:** Comparison of most important occupations

COPM (* 1 st occupations*)	Women with PMS (*n* = 38)	Women without PMS (*n* = 15)	Total	*p*
ADL	15	4	19	0.512
IADL	7	2	9	
Rest	0	1	1	
Education	9	5	14	
Leisure	2	0	2	
Social Participation	2	1	3	
Health Management	2	2	4	
Total	37	15	52	

*PMSS* Premenstrual Syndrome Scale, *COPM* Canadian Occupational Performance Measure, *ADL* Activities of Daily Living, *IADL* Instrumental Activities of Daily Living

## Discussion

This research investigated occupational performance, meaningful time use, and quality of life in women with and without PMS, and compared these parameters between the two groups. Although no direct differences in occupational performance were observed between the two groups, the time-use preferences and value perceptions of individuals with PMS exert distinct influences on occupational performance. However, the absence of significant differences in occupational scores warrants further consideration. This lack of differentiation may reflect the limited sensitivity of the COPM to short-term fluctuations associated with PMS or the possibility that participants selected problems not directly related to PMS symptoms. Acknowledging this issue would provide a more balanced interpretation of the findings and highlight the need for caution when drawing conclusions about occupational performance based solely on COPM outcomes.

In women with PMS, there was a statistically significant positive association between occupational performance and obligatory activities, and a negative association with volitional activities. In a similar vein, the perceived value of activities showed a positive relationship with obligatory activities and a negative relationship with volitional activities. Physical quality of life was negatively correlated with PMSS, while the perceived value of activities was positively correlated. Gynecological and chronic diseases may have confounding effects on PMS severity and occupational performance, and this should be taken into account in interpreting the results.

For women without PMS, a negative correlation emerged between activity value and volitional activities. Conversely, women with PMS demonstrated lower mental quality of life scores and higher activity value than their counterparts. Finally, during the premenstrual period, women with PMS identified activities of daily living as the most difficult, while women without PMS reported educational activities as the most challenging.

Hossain, Ritu, Tauhid, & Hasan [18] highlight the beneficial impact of establishing daily routines in supporting mental well-being [19] have emphasized that adherence to daily routines fosters a greater sense of psychological strength. In this cross-sectional study, correlation analysis revealed that, among women with PMS, higher engagement in obligatory activities was associated with better perceived occupational performance. Successfully completing daily routines may foster a more positive perceptual sense of mental well-being. Conversely, the lower mental quality of life scores observed in individuals with PMS indicate that mere regular engagement in daily activities may not suffice as a standalone strategy for managing PMS.

In social psychology, having a structured daily routine has been shown to serve as a fulcrum for individuals, particularly during times of distress, providing a sense of security, confidence, and meaning in life [4, 23]. The concept of routine is also central in occupational therapy literature. Historically, it has been regarded as a core principle of the discipline [13], and closely tied to the development of theoretical frameworks [5]. Necessary occupations are essential for sustaining life, and engaging in them shapes the daily routine. This routine supports self-management and continuity, while occupations performed out of obligation may further enhance occupational performance by emphasizing the importance of daily structure.

Volitional activities generally require higher motivation and better planning [5].

In our study, participants who chose to deviate from their daily routines and spend more time on volitional occupations may have been driven by a desire to enhance well-being or alleviate PMS-related complaints. To validate such choices, it is crucial to determine the personal value of these activities. Individuals primarily aim to engage in occupations they perceive as valuable. While each activity carries its own value, meaning is a broader concept. It emerges from the overall pattern of occupations across a person’s life, both in the present and over time [11, 12].

In our study, a negative correlation was observed between the increased time spent on volitional activities and the perceived value of these activities among individuals with PMS. Whether participants allocated more time to such activities with the aim of feeling better or to distance themselves from PMS related complaints cannot be determined from the present findings. However, according to the findings, during periods of PMS related symptoms, engaging in structured volitional activities that not only provide a sense of well-being but are also balanced with other meaningful obligatory activities may facilitate coping with PMS while preserving the perceived value of these activities. Although, these findings indicate associations rather than causal relationships, and alternative explanations such as the potential influence of symptom severity should be considered when interpreting these results.

Our research revealed that women with PMS primarily identified daily life activities as the main areas of difficulty during the premenstrual period, whereas women without PMS highlighted educational activities. Previous studies support these findings: [25] reported that PMS symptoms hinder occupational performance and self-care, while [10] demonstrated that PMS negatively affects daily life activities on a global scale. According to the hierarchy of needs, the continuity of daily life takes precedence over educational activities,therefore, the goals identified between the two groups may differ [33]. Furthermore, our findings suggest that individuals with PMS achieve greater success when they continue to engage in necessary daily activities. Thus, maintaining participation in daily activities may serve as an important initial intervention target for individuals with PMS. To support this, strategies such as daily planning, activity simplification, or motivational counseling may be beneficial.

In our study, qualitative analysis of the daily activity reports of individuals with PMS highlighted efforts centered on physical activity, with showering standing out as the most frequently reported activity. In contrast, individuals with PMS demonstrate a reluctance to abandon this demanding activity, a tendency that may be explained by the relaxing properties of water [3]. Accordingly, during phases of increased complaints, maintaining engagement in valued activities through structured approaches, rather than avoidance, may constitute a beneficial strategy for managing syndromes.

Our findings revealed that individuals with PMS perceived their activities as more valuable compared to those without PMS [8] emphasizes that optimal experiences in everyday life enhance overall quality of life and provide meaning. Similarly, [15] conceptualizes value as the acquisition of useful competence, where performance results in a tangible product that holds significance for the client or others. In this context, individuals with PMS may have attributed greater value to their activities due to a perceived deficiency in the meaningful performance derived from their daily routines. Although women with PMS reported significantly higher MOQ Value scores, this direction of association is not necessarily obvious. It may reflect complex cognitive appraisal processes, such as effort justification [17], whereby greater effort invested in activities during symptomatic periods enhances their perceived value [30]. Alternatively, this finding may be sample-specific or due to chance, and should therefore be interpreted cautiously in light of prior literature.

Our findings, consistent with previous studies [2, 20, 26], demonstrate that PMS has a significant impact on women’s quality of life. In our study, women with PMS exhibited lower mental quality of life compared to those without PMS. Moreover, as the severity of symptoms increased, physical quality of life showed a marked decline. This suggests that women experiencing severe symptoms during the premenstrual period may encounter substantial difficulties in fulfilling daily responsibilities due to the adverse effects on their physical health.

Importantly, the influence of PMS extends beyond physical health to psychosocial functioning. Women may experience changes in their activity preferences, performance, and the value they attribute to these activities, largely due to the psychological and mental complexity associated with the premenstrual phase. Therefore, PMS should be regarded as a multidimensional condition that affects both physical and mental aspects of quality of life. These findings underscore the necessity of considering PMS not only in terms of symptom management but also in relation to the preservation and enhancement of overall quality of life in clinical practice.

This study should be evaluated comprehensively, taking into account its strengths and limitations. The evaluation of women during their premenstrual periods is a strength of the study. In addition, the fact that a 24-h day of the participants was analyzed is another strength. However, one limitation of this study is that it was completed with a relatively small number of participants, the majority of whom experienced PMS. Therefore, it is essential to consider this circumstance when interpreting the results. Another limitation is that different parameters in the lives of women that we did not evaluate may also affect the results. Furthermore, determining menstrual periods based on personal calendars and menstrual cycles rather than hormonal tests may lead to misclassifications. Finally, the narrow age range of participants may influence occupational preferences, making it difficult to generalise for all women with PMS.

## Conclusion

To the best of our knowledge, this study is the first to evaluate the impact of PMS on occupational performance and meaningful time use. The findings of this study indicate that increasing the time allocated to daily routine activities in women experiencing PMS enhances occupational performance level and perceived value of occupations. However, increased severity of PMS is associated with a decline in women's physical quality of life. Furthermore, improvements in quality of life appear to be positively affect the value attributed to activities. Another noteworthy finding of the study is that women with PMS have a lower mental quality of life and give more importance to their activities compared to women without PMS. These results highlight the complicated interaction between PMS severity, occupational performance, and meaningful time use. Further research is necessary to clarify the processes behind these relationships and to design rehabilitation programs for developing the quality of life for women with PMS.

## Data Availability

The datasets presented in this article are not readily available due to restrictions (e.g., their containing information that could compromise the privacy of research participants). Requests to access the datasets should be directed to İYT, ibrahimyavuz.tatli @ksbu.edu.tr.

## Abbreviations

PMS: Premenstrual syndrome
COPM: Canadian occupational performance measure
MOQ: Modified occupational questionnaire
SF-12: 12-Item short-form health survey
PMSS: Premenstrual syndrome scale
